# Short-Term Effects of Orthokeratology Treatment Zone Decentration on Choroidal Thickness and Vasculature

**DOI:** 10.1167/tvst.15.4.1

**Published:** 2026-04-06

**Authors:** Liyuan Yang, Weiping Lin, Tongtong Jia, Ruihua Wei

**Affiliations:** 1 Tianjin Key Laboratory of Retinal Functions and Diseases, Tianjin Branch of National Clinical Research Center for Ocular Disease, Eye Institute and School of Optometry, Tianjin Medical University Eye Hospital, Tianjin, P.R. China; 2 Department of Ophthalmology and Optometry, Gansu Provincial Hospital, Lanzhou, Gansu, China

**Keywords:** myopia control, orthokeratology, treatment zone decentration, choroid

## Abstract

**Purpose:**

This study investigated the short-term effects of orthokeratology (ortho-K) treatment zone decentration (TZD) on choroidal thickness (ChT) and vasculature in children with myopia.

**Methods:**

Children aged 8 to 12 years with myopia undergoing ortho-K were recruited from the Tianjin Medical University Eye Hospital. Subfoveal ChT, choroidal vascularity index, and choriocapillaris perfusion area were assessed using optical coherence tomography and optical coherence tomography angiography at baseline and after 1 month of lens wear. Corneal topography was used to quantify the TZD.

**Results:**

After 1 month of ortho-K, ChT significantly increased by 17.74 ± 8.89 µm and 18.25 ± 8.98 µm in the low and moderate myopia groups, respectively (*P* < 0.05). The choriocapillaris perfusion area also increased by 0.013 ± 0.014 mm^2^ and 0.017 ± 0.017 mm^2^, respectively, although no significant change was observed in the choroidal vascularity index. Multiple regression analysis revealed that changes in ChT were significantly correlated with baseline spherical equivalent and the magnitude of the TZD (all *P* < 0.05). However, there was no significant correlation between the direction of decentration and quadrant-specific changes in ChT.

**Conclusions:**

A larger TZD was significantly associated with a greater increase ChT after 1 month of ortho-K treatment, particularly in children with relatively high myopia.

**Translational Relevance:**

Orthokeratology treatment zone decentration is associated with choroidal thickening during 1-month orthokeratology treatment. This early change may provide a new perspective for evaluating its potential role in long-term myopia control.

## Introduction

The rapid increase in myopia among children and adolescents has become a global public health concern. Currently, myopia affects approximately 34% of the global population, and this prevalence is projected to reach 50% by 2050, particularly in East Asia.^1^^,^^2^ Progressive myopia greatly increases the risk of vision-threatening complications, including myopic maculopathy, choroidal neovascularization, retinal detachment, and posterior scleral staphyloma.^3^^,^^4^

Accumulating evidence suggests that the choroid plays an important role in myopia progression, as demonstrated in both animal models and human studies.^5^^,^^6^ Myopia severity is correlated with changes in choroidal thickness (ChT), with high myopia inducing greater ChT thinning and reduced choroidal perfusion.^7^

Orthokeratology (ortho-K), a widely adopted intervention for myopia control, has been shown to decrease axial elongation by 32% to 55%.^8^^,^^9^ Factors such as age at initiation, baseline spherical equivalent, axial length, and pupil diameter have been found to influence the efficacy of myopia control; however, the underlying mechanisms remain unclear.^10^^,^^11^ Recent studies have shown that ortho-K lens wear increases ChT and choroidal vascularity, with less ocular elongation.^12^

Lin et al.^13^ reported that treatment zone decentration (TZD) of ortho-K lenses can delay myopia progression. However, the effects of TZD on the choroid have not been investigated thoroughly. Whether TZD influences ChT and vasculature requires further investigation. This study aimed to evaluate the effects of TZD on ChT and choroidal vasculature in children undergoing ortho-K treatment, providing insights into the mechanism of myopia control after ortho-K.

## Methods

### Participants

This prospective study was conducted at Tianjin Medical University Eye Hospital (Tianjin, China) between January and March 2025. Patients who met the criteria were enrolled in this study. The inclusion criteria were as follows: age between 8 and 15 years; spherical equivalent (SE) from −0.75 to −6.00 D; astigmatism of ≤1.5 D; and best-corrected visual acuity of ≥20/20. The exclusion criteria included active ocular inflammation or ocular surface disease, strabismus, a history of ocular surgery, and prior use of myopia control methods, such as 0.01% atropine eye drops, multifocal contact lenses, and repeated low-level red light. Patients were stratified into two groups: the low myopia (LM) group (−3.00 D < SE ≤ −0.50 D) and the moderate myopia (MM) group (−6.00 D < SE ≤ −3.00 D).^14^^,^^15^

### Ethical Approval Statement

This study adhered to tenets of Declaration of Helsinki and was approved by Ethics Committee of Tianjin Medical University Eye Hospital (2025KY-05).

### Patient Consent Statement

All participants and their guardians provided written informed consent after a detailed explanation of the ortho-K lens procedure was provided.

### Ortho-K Lens Fitting

All patients were fitted with four-zone reverse geometry ortho-K lenses (iBright), with an oxygen permeability of 125 × 10^−11^ (cm^2^/s) (mLO_2_/mL·mm Hg), a nominal central thickness between 0.20 and 0.25 mm, back optical zone diameter of 5.8 to 6.2 mm, and base curve from 8.44 mm (40.00 D) to 7.42 mm (46.00 D). Lens fitting followed standard clinical guidelines. The initial trial lens was selected based on corneal flat K, astigmatism, and eccentricity. An ideal fit was defined as a centrally located optical zone covering the pupil, absence of lens decentration, and lens movement of <1 mm. The Jessen Factor power was set to +0.75 D.

Patients were instructed to wear their ortho-K lenses for ≥8 consecutive hours per night. They received comprehensive training on lens handling and hygiene. At each follow-up, all patients underwent a thorough examination, which include slit-lamp examination, visual acuity, and corneal topography.

### Ocular Biometric Measurements

All participants underwent a comprehensive baseline examination. Myopic refractive error was determined by cycloplegic refraction using compound tropicamide eye drops (5 mg/mL, one drop every 5 minutes, four times). Axial length was measured using the IOL-Master 500 (Zeiss, Jena, Germany), and the average value was analyzed.

### Treatment Zone Size (TZS) and TZD

**Figure 1.**
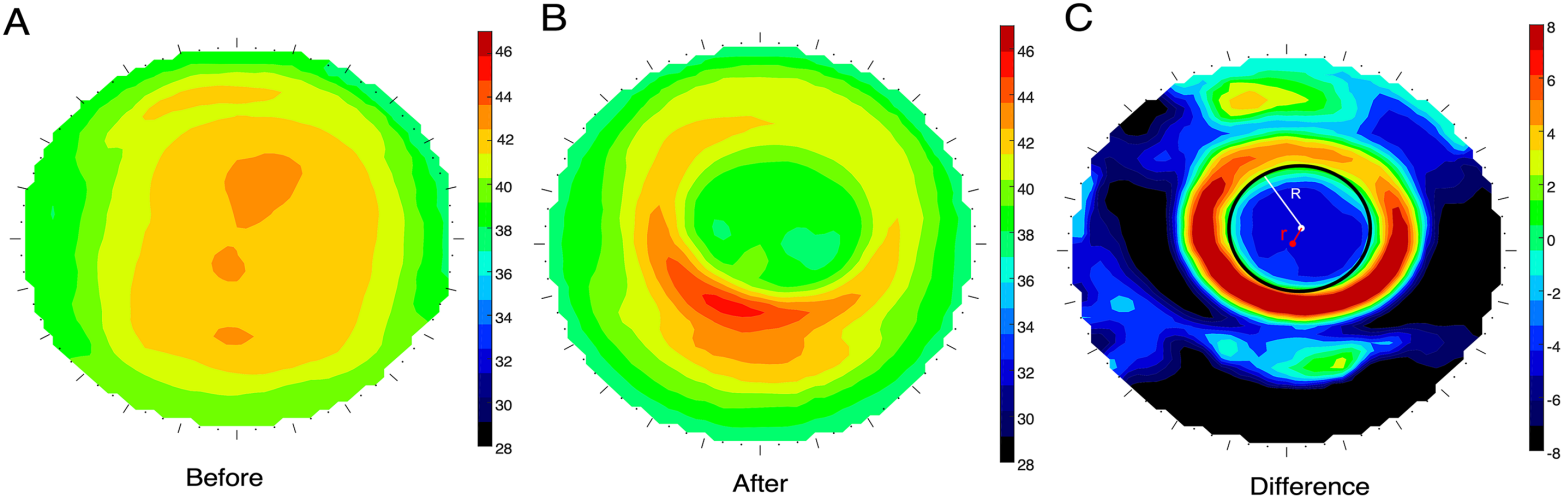
Measurement of the TZD and TZS. (**A**) Tangential map at baseline. (**B**) Tangential map at 1 month post treatment. (**C**) Difference map used to quantify the TZD and TZS. The *red* and *white dots* represent the corneal apex and center of the treatment zone, respectively. The *black circle* indicates the boundary of the treatment zone. The distance between the white and red dots represents the decentration of the treatment zone (TZD). The radius of the *black circle* was defined as the TZS. R, radius of treatment zone circle; r, the distance of TZD.

Corneal topography was performed three times at each follow-up visit using the TMS-4 system (Tomey, Tokyo, Japan) by a single experienced technician (Figs. 1A, 1B). The best-focused topography map (with the accuracy >95%) was selected from the automatically captured images. Each map consisted of 31 rings, with 256 data points per ring. The TZS and decentration were calculated as described previously.^13^ Corneal difference maps were calculated by subtracting the 1-month post-treatment tangential curvature map from the baseline map to quantify the TZD. The central and paracentral regions within radius 4mm where locations decreased by more than 0.00 D were identified as the treatment zone, and its boundary was fitted into a circle using a custom MATLAB function (MathWorks, Natick, MA) (Fig. 1C). The distance between the center of the cornea and the center of the treatment zone was defined as the TZD.^16^

### Swept-source Optical Coherence Tomography (OCT)/OCT Angiography Scanning and Analysis

The swept-source OCT/OCT angiography system VG200S (SVision Imaging, Henan, China), with a central wavelength of 1050 nm and a scan rate of 200,000 A-scans per second, was used to assess the choroid structures and vascular characteristics. To minimize the potential confounding effects of diurnal variations, all measurements were conducted between 12 pm and 3 pm. The system was equipped with an eye-tracking device to minimize motion artifacts.

**Figure 2.**
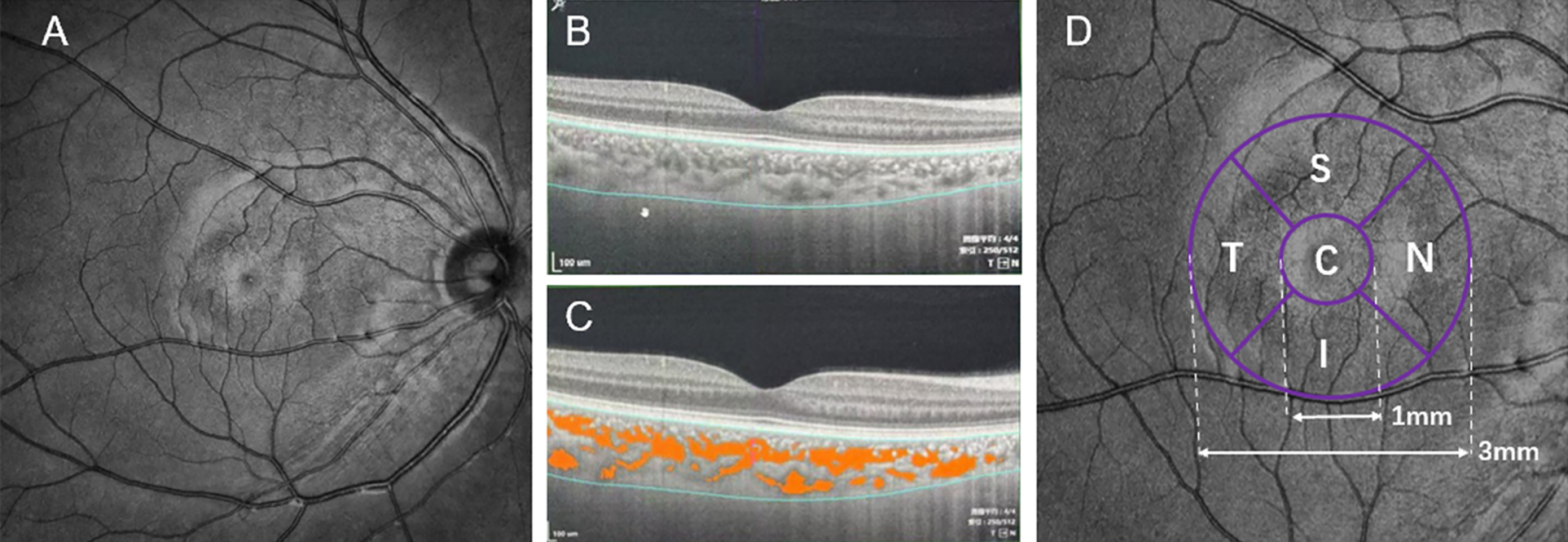
Analysis of choroidal structure and vasculature. Measurement of the subfoveal ChT and CVI. (**A**) Structural OCT scan, (**B**) measurement of ChT at the central fovea, defined as the distance between the retinal pigment epithelium–Bruch's membrane complex and the choroid–sclera interface. (**C**) The system recognizes the choroidal boundaries and identifies the large and medium choroidal blood vessels (*red*) and stromal areas (grey) for CVI calculation. (**D**) Macular Early Treatment Diabetic Retinopathy Study grid. C, center; I, inferior; N, nasal; S, superior; T, temporal.

The three-dimensional angiographic data were obtained using a raster scan protocol of 512 continuous horizontal and vertical B-scans covering a of 6 × 6 mm area centered on the fovea (Fig. 2A). The ChT and vascular parameters were automatically segmented and measured using the built-in software of the OCT instrument. Segmentations were also corrected manually for segmentation errors.^17^

**Figure 3.**
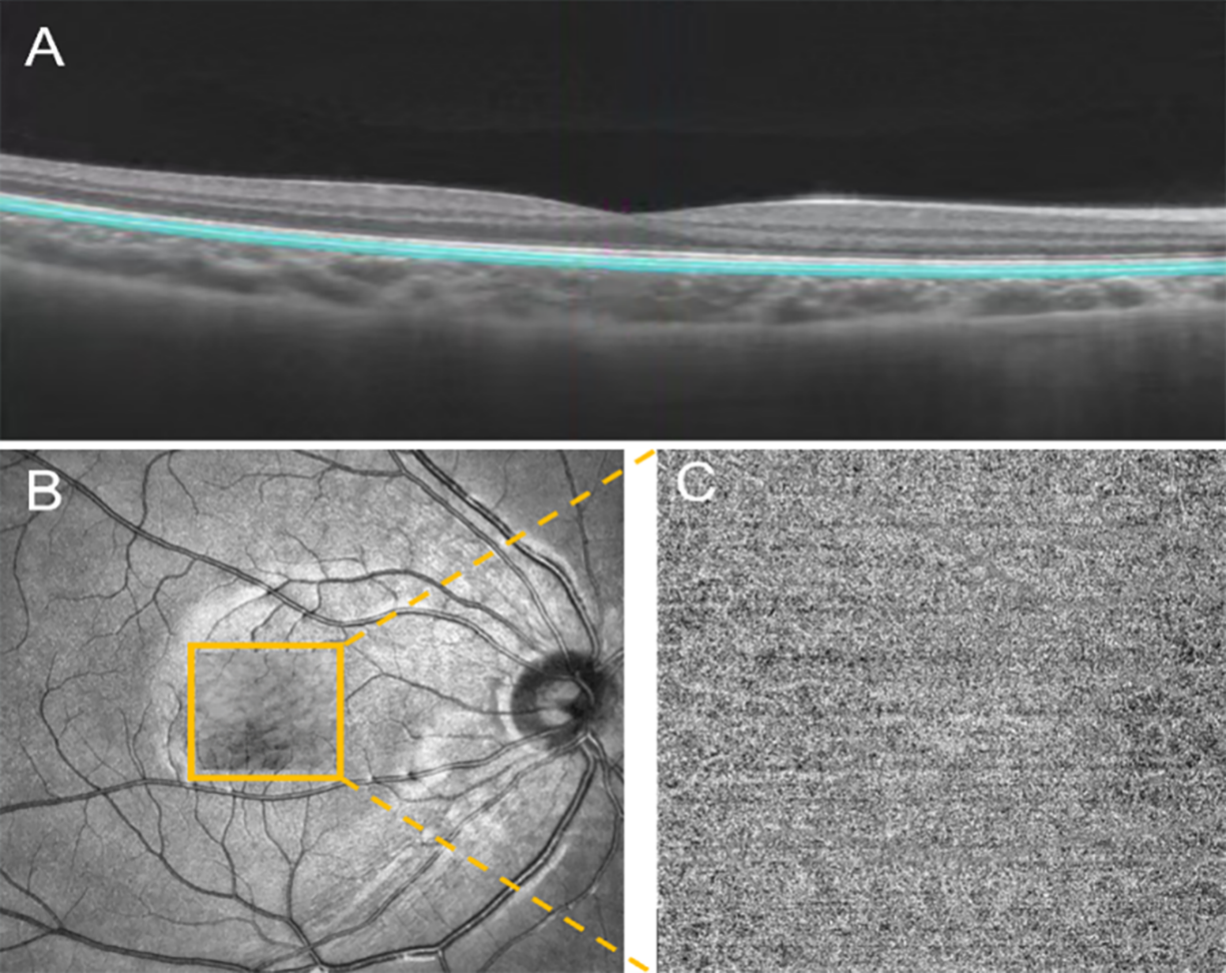
Choriocapillaris blood perfusion analysis. (**A**) Structure of the choriocapillaris, (**B**) 3 × 3 mm region of the OCT-A scan, and (**C**) magnified en face OCT-A choriocapillaris image. OCT-A, OCT angiography.

ChT centered on the fovea was defined as the distance between the retinal pigment epithelium–Bruch's membrane complex and the choroid–sclera interface (Fig. 2B). The choroidal vascularity index (CVI) was defined as the ratio of the choroidal luminal area to the total choroidal area (Fig. 2C). The choriocapillaris slab was defined as the region extending 20 µm below the retinal pigment epithelium–Bruch's membrane complex (Fig. 3A).^18^ The choriocapillaris perfusion area (CCPA), which estimates choroidal blood perfusion, was calculated automatically by the system software (Fig. 3C). The scanning size was adjusted according to the differences in fundus magnification caused by different axial lengths of the eyes. By using a scaling factor formula of 0.0492 × Axial length − 0.1818,^19^^,^^20^ the OCT device can correct fundus images and eliminate magnification disparities. These parameters were measured three times and averaged to ensure data reliability.

According to the Early Treatment Diabetic Retinopathy Study grid, the macular zone was automatically partitioned into circular concentric areas of 1 mm and 3 mm. The annulus was further subdivided into superior, inferior, temporal, and nasal quadrants (Fig. 2D).

### Statistical Analysis

All statistical analyses were performed using SPSS version 26.0 (IBM Corp., Armonk, NY). The Kolmogorov–Smirnov test was used to assess normality of distribution, followed by a test for homogeneity of variances. Data with normal distributions were presented as mean ± standard deviation, and data with non-normal distributions were presented as median and interquartile range. Analysis of variance was used for normally distributed categorical data, while the Mann–Whitney *U* test was used for non-normally distributed data. Univariate linear regression was used to analyze the relationships among ChT, TZS, and TZD. Multiple linear regression analyses were used to analyze the relationships between ChT and baseline age, baseline ocular biometrics, TZS, and TZD. Statistical significance was set to a *P* value of <0.05.

## Results

**Figure 4.**
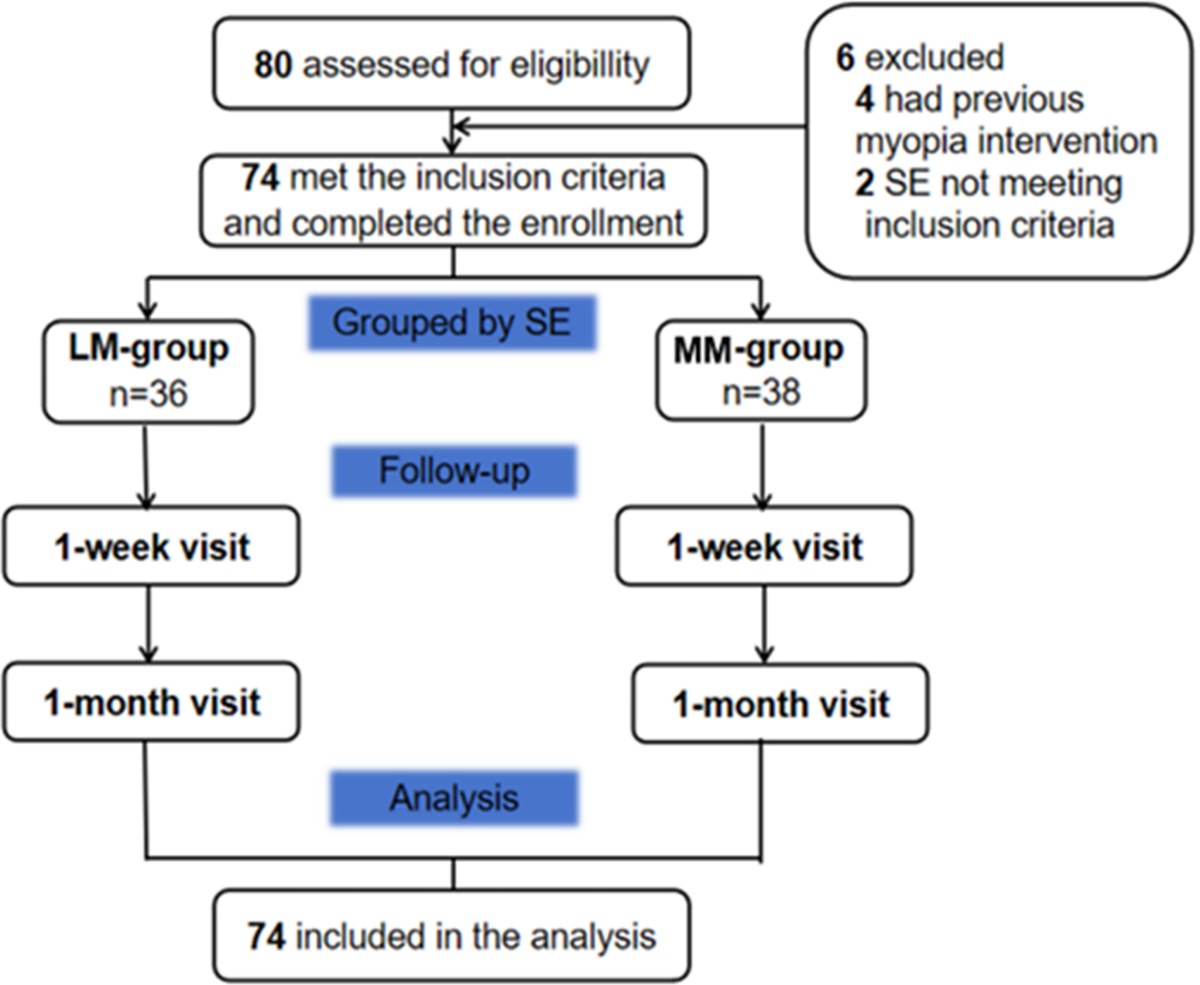
Flow diagram showing the trial profile.

A total of 74 participants (74 eyes) were enrolled and completed the follow-up examinations (Fig. 4). The mean age was 10.01 ± 1.28 years (range, 8–12 years). At baseline, the mean SE, axial length, corneal flat K, and corneal steep K were −3.44 ± 1.48 D (range, −6.00 to −0.75 D), 24.88 ± 0.88 mm, 42.95 ± 1.23 D, and 44.17 ± 1.23 D, respectively. Only data from the right eye were used for the statistical analyses.

The ChT at baseline was significantly greater in the LM group than in the MM group. No significant differences were observed in the other baseline ocular and choroidal metrics between the two groups (Table 1).

**Table 1.** Baseline Characteristics

Characteristic	Total (*n* = 74)	LM Group (*n* = 36)	MM Group (*n* = 38)	*P* Value
Demographics				
Age (years)	10.01 ± 1.28	9.98 ± 1.29	10.02 ± 1.21	0.43
Sex (male/female)	40/34	18/18	22/16	0.32
Ocular biometrics				
SE (D)	−3.44 ± 1.48	−2.22 ± 0.61	−4.57 ± 1.09	<0.001^*^
AL (mm)	24.88 ± 0.88	24.36 ± 0.68	25.33 ± 0.78	<0.001^*^
K flat (D)	42.95 ± 1.23	42.94 ± 1.19	43.05 ± 1.32	0.53
K steep (D)	44.17 ± 1.23	44.15 ± 1.23	44.21 ± 1.25	0.94
Choroidal metrics				
ChT (µm)	263.97 ± 54.47	285.55 ± 50.82	251.29 ± 54.17	0.006^*^
CVI CCPA (mm^2^)	0.45 ± 0.08	0.46 ± 0.09	0.43 ± 0.07	0.59
	0.72 ± 0.03	0.73 ± 0.03	0.71 ± 0.02	0.43

AL, axial length; K, corneal keratometry.

Data are presented as mean ± standard deviation.

* *P* < 0.05.

### TZD and TZS

TZD exhibited a non-normal distribution, whereas TZS was normally distributed. After treatment, the median TZD was 0.28 mm (range, 0.04–1.28 mm), and the mean TZS radius was 2.71 ± 0.46 mm (range, 1.57–3.74 mm). The TZD was 0.23 mm (range, 0.14–0.40) in the LM group, compared with 0.35 mm (range, 0.23–0.60) in the MM group (*P* < 0.01). The mean TZS in the LM and MM groups was 2.85 ± 0.33 and 2.62 ± 0.53 mm, respectively (*P* < 0.01) (Table 2).

**Table 2.** Comparison of TZD and TZS Between the LM Group and MM Group

	TZD (mm)	TZS (mm)
LM group	0.23 (0.14–0.40)	2.85 ± 0.33
MM group	0.35 (0.23–0.60)	2.61 ± 0.53
*P* value	0.01^*^	0.02^*^

Normally distributed data are presented as mean ± standard deviation, and non-normally distributed data are presented as median (interquartile range).

* *P* < 0.05.

**Table 3.** Multivariable Regression Analysis Showing The Association 1-Month Changes in ChT and Two Variables: The Baseline SE and TZD

Variables	Beta	*P* Value	95% Confidence Interval
Baseline SE	1.504	0.03^*^	0.172–2.837
TZD	18.841	<0.001^*^	12.496–25.188
Model	*R* ^2^ = 0.326		

* *P* < 0.05.

### Choroidal Changes Following Ortho‑K Wearing

**Figure 5.**
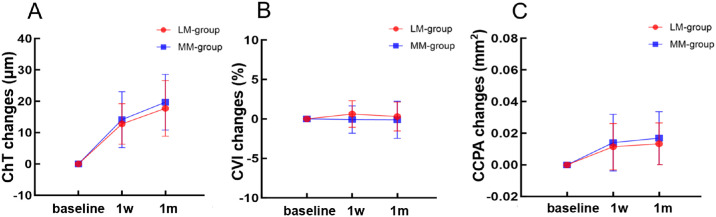
Changes of Choroidal structure and vasculature during 1 month of follow-up. (**A**) ChT, (**B**) CVI, and (**C**) CCPA.

After 1 month of ortho-K treatment, significant changes in choroidal structure and vasculature were observed. In the LM and MM groups, ChT significantly increased by 12.77 ± 6.43 and 13.92 ± 8.66 µm, respectively, at 1 week, and by 17.74 ± 8.89 and 18.25 ± 8.98 µm, respectively, at 1 month (Fig. 5A). In the LM and MM groups, CCPA increased by 0.011 ± 0.015 mm^2^ and 0.015 ± 0.018 mm^2^, respectively, at 1 week, and by 0.013 ± 0.014 mm^2^ and 0.017 ± 0.017 mm^2^, respectively, at 1 month (Fig. 5C). However, no significant changes were observed in the CVI at the 1-week and 1-month follow-up.

### Association Between 1-Month Choroidal Changes and TZD

**Figure 6.**
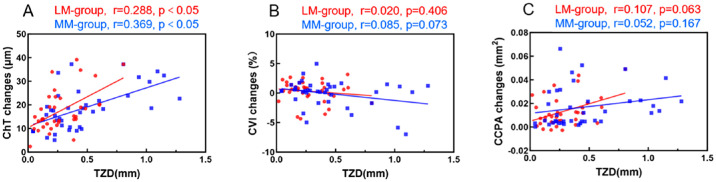
Correlation between 1-month changes in the choroidal metrics and TZD. (**A**) ChT, (**B**) CVI, and (**C**) CCPA.

Univariate linear regression analysis revealed a significant positive correlation between the TZD and ChT at 1 month (*P* < 0.05). In contrast, no significant associations were observed between the TZD and the CVI or CCPA (Fig. 6).

### Multiple Regression for 1-Month ChT Changes and Ocular Biometrics

A multiple regression analysis was conducted between 1-month ChT changes and ocular biometrics including baseline age, baseline SE, baseline K-value, baseline axial length, TZD, and TZS. The analysis showed that the change in ChT was significantly correlated with baseline SE (*t* = 2.25; *P* = 0.03) and TZD (*t* = 5.92; *P* < 0.001) (*R*^2^ = 0.32), but not with the other factors being analyzed (Table 3).

### ChT Changes of Four Quadrant, Ortho-K Treatment Decentration Direction and the Correlation

**Figure 7.**
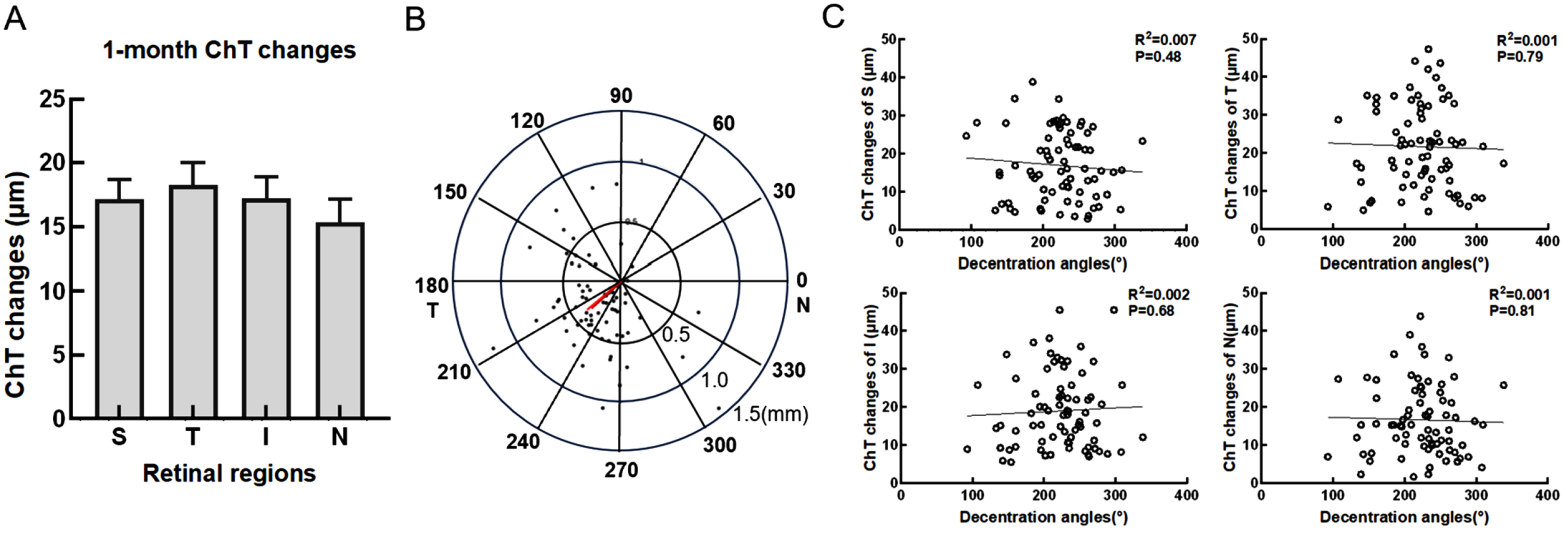
ChT changes of four quadrant, ortho-K treatment decentration direction and the correlation. (**A**) Changes in ChT. (**B**) The distribution of decentration direction. The red line represent the direction and magnitude of the decentration. (**C**) The correlation between the changes in ChT of four quadrant and the decentration direction.

After 1 month of ortho-K treatment, the changes of ChT in the four quadrants were: superior, 17.12 ± 13.86 mm; inferior, 18.28 ± 14.91 mm; temporal, 17.22 ± 14.68 mm; and nasal, 15.35 ± 14.39 mm. There was no statistically significant difference in the changes of ChT in the four quadrants. Most patients had treatment decentration directed toward the inferior temporal quadrant, with a mean amount of decentration of 0.37 ± 0.28 mm and direction of 219.45 ± 45.3°. A correlation analysis revealed no significant associations between decentration direction and the ChT changes of four quadrants at 1 month (Fig. 7).

## Discussion

In this study, ortho-K TZD was associated with increased ChT in children undergoing ortho-K treatment, especially among those with relatively high myopia. Multiple linear regression analysis revealed that larger TZD correlated with greater ChT thickening.

Increasing evidence suggests that ortho-K lens decentration decreases myopia progression.^21^^,^^22^ The retinal–choroid–sclera signaling pathway has been implicated in the progression of myopia.^23^^,^^24^ Because decentration retards the progression of myopia, we investigated whether it also influences the choroidal structure. This study focused on choroidal changes in children with myopia wearing ortho-K lenses and the correlation between TZD and these choroidal changes.

### Distribution of the TZD and TZS

The TZD and TZS have been underexplored compared with established myopia control factors such as age and SE.^9^^,^^25^ Previous studies have shown that a smaller TZS and a larger TZD are associated with reduced axial elongation.^13^^,^^22^ In the current study, at the 1-month follow-up, TZD was 0.28 mm (range, 0.04–1.28 mm), consistent with a previous study by Lin et al., who reported a mean TZD of 0.52 ± 0.22 mm (range, 0.05–1.24 mm) in 352 subjects.^13^

Our study demonstrated that the most common direction of decentration was inferotemporal, followed by supertemporal and temporal decentration.^26^ Similarly, Chen et al.^27^ reported 84.9% temporal decentration in 106 eyes, whereas Yang et al.^28^ reported temporal decentration in 48.5% of 270 eyes. This trend may be due to the relatively elevated nasal side compared with the temporal side after ortho-K treatment, causing the lens to shift temporally. Considering that lens decentration is difficult to avoid in clinical practice, it is important to ensure that any decentration remains within a clinically acceptable range that does not induce visual discomfort, such as glare, ghosting, and corneal epithelial injury.^26^^,^^29^ Hence, deliberate decentration of ortho-K lenses is not recommended.

### Choroidal Changes After Ortho-K Wear

Previous studies have reported that the ChT and vascular characteristics are altered during the early stages of ortho-K treatment.^30^^,^^31^ Notably, greater decentration has been associated with improved efficacy in preventing axial elongation in myopic children wearing ortho-K lenses.^32^ Recent studies have shown that a greater ChT thinning and a decrease in choroidal vasculature, as assessed by the CVI, are associated with more severe degrees of myopia.^33^^,^^34^

The current study showed that the ChT significantly increased in both the LM and MM groups after 1 month of ortho-K treatment, with no significant difference in the magnitude of change between the two groups. Additionally, the CCPA also increased in both the LM and the MM groups, and the CVI remained stable. Previous studies have also demonstrated significant ChT thickening after 1 month of wearing ortho-K lenses, consistent with our findings.^11^ Li et al.^30^ reported that the change in ChT peaked at 1 month and persisted over 1 year of follow-up period. Several studies have reported that peripheral myopic defocus and higher-order aberrations play a role in controlling myopia.^35^^,^^36^ The increase in myopic defocus and higher-order aberrations with lens decentration may explain the greater ChT thickening. Further studies are needed to determine whether TZD will cause greater defocus or increase higher-order aberrations.

### Association Between Choroidal Changes and the TZD

Correlation analyses revealed that a larger TZD was significantly associated with greater ChT thickening. No significant correlations were found between the TZD and the CVI or CCPA. Although the ChT was positively correlated with the TZD in both the LM and the MM groups, the correlation was relatively low in the LM group. After adjusting for confounding factors such as age, multivariate regression analysis confirmed that the TZD was an independent predictor of changes in ChT. These findings suggest that the effect of decentration on choroidal response may vary by myopia severity, which may guide clinical strategies for ortho-K lens fitting. Monitoring trends in ChT during early ortho-K wear may help to identify children who are more likely to benefit from treatment.^37^

### Potential Mechanism

Ortho-K has achieved 32% to 55% efficacy in controlling myopia progression in children, although the efficacy of myopic control varies among individuals,^38^ and the underlying mechanisms are yet to be explored. Increased ChT and blood flow may precede and mitigate scleral hypoxia and remodeling, ultimately slowing myopia progression.^39^ Studies have revealed that the choroid is involved in relaying visual signals from the retina to the sclera, mediating axial growth, and controlling myopia. ChT typically increases by approximately 10 to 20 µm after 1 month of ortho-K treatment and stabilizes at 1 year.^12^ Cho et al.^40^ speculated that a greater corneal reshaping effect enhances peripheral myopic defocus and improves myopia control efficacy.

We hypothesized that an increase in the ChT and the CCPA may enhance choroidal oxygenation and circulation, modulating scleral extracellular matrix synthesis and remodeling. Our findings revealed that the ChT significantly increased after 1 month of ortho-K treatment, with greater ChT thickening associated with a larger TZD. This early change may provide a new perspective for evaluating its potential role in long-term myopia control.

### Limitations

This study has several limitations. First, the relatively small sample size may have affected the accuracy of the results. Second, the follow-up duration was relatively short, and it is unclear whether participants experienced similar long-term changes. Finally, only a limited number of choroidal parameters were evaluated. Further studies with larger sample sizes, longer follow-up durations, and a more comprehensive evaluation of other choroidal parameters are needed to explore the impact of decentration on retinal defocus and the possible mechanisms for choroidal changes.

## Conclusions

Ortho-K TZD is associated with choroidal thickening during 1-month ortho-K treatment. This early change may provide a new perspective for evaluating its potential role in long-term myopia control.
